# Recovery of pelvic floor muscle strength and endurance 6 and 12 months postpartum in primiparous women—a prospective cohort study

**DOI:** 10.1007/s00192-022-05334-y

**Published:** 2022-09-01

**Authors:** Kari Bø, Karoline Næss, Jette Stær-Jensen, Franziska Siafarikas, Marie Ellström Engh, Gunvor Hilde

**Affiliations:** 1 Department of Sports Medicine, Norwegian School of Sport Sciences, PO Box 4014, Ullevål stadion, 0806 Oslo, Norway; 2 Department of Obstetrics and Gynaecology, Akershus University Hospital, Lørenskog, Norway; 3 Department of Health, Care and Welfare, Ullensaker municipality, Ullensaker, Norway; 4 Department of Physiotherapy, Faculty of Health Sciences, OsloMet – Oslo Metropolitan University, Oslo, Norway; 5 Faculty Division Akershus University Hospital, University of Oslo, Oslo, Norway

**Keywords:** Endurance, Pelvic floor muscles, Postpartum, Recovery, Strength

## Abstract

**Introduction and hypothesis:**

To date there has been scant knowledge on the natural recovery of the pelvic floor muscles (PFMs) after childbirth. The aims of the present study were to investigate whether PFM variables at 6 and 12 months postpartum had returned to mid-pregnancy levels and assess risk factors for reduced recovery at 12 months postpartum.

**Methods:**

This was a prospective cohort study following 235 nulliparous pregnant women from mid-pregnancy to 12 months postpartum. Vaginal resting pressure (VRP), PFM strength and endurance were assessed by manometry at 22 weeks, 6 and 12 months postpartum. Multiple linear regression was used to address factors influencing PFM variables beyond birth mode.

**Results:**

Cesarean section was protective for change in PFM variables. From mid-pregnancy to 12 months postpartum there was a 20% reduction in VRP (*p*<0.001) and a 7.5 % reduction in PFM strength (*p*=0.007), and an increase of 9% in PFM endurance (*p*=0.002) in the normal vaginal birth. The instrumental vaginal group had a decline in VRP of 21% (*p*<0.001) and PFM strength of 15% (*p*=0.011), but no significant change in PFM endurance. Higher BMI at 12 months postpartum, longer second stage of labor, and major tears of the levator ani muscle had a negative influence on the PFM recovery beyond delivery mode.

**Conclusions:**

At 12 months postpartum following vaginal delivery, the PFMs are not fully recovered compared with mid-pregnancy values. More follow-up physical therapy may be warranted in the postpartum period, especially for women with complicated vaginal births and higher BMI.

## Introduction

Vaginal birth, especially instrumental delivery, is established as a risk factor for the development of pelvic floor dysfunction (PFD) such as urinary incontinence (UI), anal incontinence (AI), and pelvic organ prolapse (POP) [1–4]. Reduced function of the pelvic floor muscles (PFM) due to childbirth may contribute to PFD [5]. Vaginal resting pressure (VRP) and PFM strength and endurance can be measured by vaginal palpation, manometry, and dynamometry [6], but to date there has been a paucity of longitudinal studies with measurement of these variables in nulliparous pregnant women from pregnancy into the postpartum period. As pregnancy and childbirth may negatively affect PFM function [2], it is important to assess recovery of the PFM during this period.

We have previously reported longitudinal data on PFM variables assessed by manometry of 277 first-time pregnant women assessed at gestational week (GW) 22 and 6 weeks postpartum [7]. At 6 weeks postpartum women with normal vaginal delivery had a reduction in VRP, PFM strength, and PFM endurance of 29%, 54%, and 53% respectively. PFM strength was significantly more reduced after instrumental versus non-instrumental vaginal delivery, and vaginal delivery had a significantly greater influence on VRP and on PFM strength and endurance than cesarean section (CS). The CS group had no change in PFM strength or endurance, but a reduction in VRP by 10% [7]. In a cross-sectional study, we compared PFM variables in women with and without major levator ani tears 6 weeks postpartum and found no difference in VRP between the two groups, but PFM strength and endurance were 47% lower in those with major tears than in women with intact PFMs [8]. To date we have only been able to find one longitudinal study presenting clinical data on VRP and PFM strength and endurance from pregnancy to 12 months postpartum, and the knowledge of natural recovery of injured and intact PFMs in the 1st year postpartum is therefore sparse [9].

The aims of the present study were to:

1. Investigate whether VRP and PFM strength and endurance at 6 and 12 months postpartum had returned to mid- pregnancy levels in women with normal vaginal delivery, instrumental vaginal delivery, and CS
2. Assess the influence of demographic and obstetric variables on recovery of VRP and PFM strength and endurance at 12 months postpartum

## Materials and methods

### Design

This is a prospective cohort study including 300 nulliparous women scheduled for delivery at Akershus University Hospital, Norway [7, 10]. The study was approved by the Regional Medical Ethics Committee (REK South East 2009/170) and Norwegian Social Science Data Services (2799026) and was registered at ClinicalTrials.gov (NCT01045135). All participants gave written informed consent before entering the study.

### Participants

Nulliparous women were recruited in gestational weeks 18–22 (mid-pregnancy) in their first pregnancy and were followed until 12 months postpartum. Inclusion criteria were a singleton pregnancy and being able to speak and understand a Scandinavian language. Women with a prior delivery (miscarriage) after gestational week 16 and serious maternal or fetal pathological conditions were excluded. To attend the study visits postpartum, the women had to give birth after GW 32. Women with stillbirth were excluded. At 6 weeks postpartum 71 women within the cohort participated in the training arm of a 4-month randomized controlled trial (RCT) evaluating the effect of pelvic floor muscle training (PFMT) on UI [11]. There was no effect of the postpartum PFMT in this RCT, neither on PFM variables nor on UI, and we therefore included them in the present analysis. For the research questions of the present study only women with intact dataset of PFM variables at mid-pregnancy and at 6 months and 12 months postpartum were included. The sample size of 300 participants was based on power calculation for expected change in levator hiatus (LH) dimensions (transperineal ultrasound) from pregnancy to postpartum [10]. No further power calculations were done for the present study.

### Data collection

Demographic data were collected through an electronic questionnaire in connection with the participants’ clinical examinations at gestational week 18–22 (mid-pregnancy). Data on delivery mode and other obstetric variables were collected from the hospital’s electronic birth records (PARTUS). Data on PFM training (self-report) were collected using an electronic questionnaire at mid-pregnancy and at 6 months and 12 months postpartum.

Major tears of the levator ani muscles were assessed by transperineal ultrasound 6 weeks postpartum using the GE Kretz Voluson E8 (GE Healthcare AS, Oslo, Norway) with a 4- to 8-MHz curved array 3D/4D ultrasound transducer (RAB4-81/obstetric). Major defects of the levator ani muscle were assessed using tomographic imaging of the axial plane at maximal PFM contraction and diagnosed according to Dietz et al. [12, 13]. The method has shown good intra- and inter-rater reliability shortly after childbirth [14]. The gynecologists performing the ultrasound assessments were blinded to delivery mode during examinations in the postpartum period.

### PFM measurements

At the first visit, participants were taught how to perform a correct PFM contraction. PFM contraction without any movement of the pelvis or visible contraction of the gluteal, hip, or abdominal muscles was emphasized [15]. All examinations were performed with the participants in a standardized supine crook lying position. Correct contraction was assessed by visual observation and vaginal palpation and defined as an inward movement and squeeze around the pelvic floor openings [15, 16].

The VRP and PFM strength and endurance were measured using an air-filled vaginal balloon connected to a high-precision pressure transducer (Camtech AS, Sandvika, Norway). At atmospheric pressure, the balloon was set to 0 cm H_2_O for each subject before it was placed into the vagina. The middle of the balloon was positioned 3.5 cm inside the introitus [17]. VRP was measured with the balloon positioned in the vagina without any voluntary PFM activity. PFM strength was calculated as the mean of three maximal voluntary contractions (MVCs). The method has been found to be reliable and valid if used with simultaneous observation of inward movement of the perineum/catheter during the contraction [15, 18, 19]. PFM endurance was defined as a sustained maximal contraction and was quantified during the first 10 s as the area below the measurement curve [20]. Two physical therapists conducted the measurements. To minimize biases in the assessment and manometer measurements, the assessors were trained ahead of the study, and a rigorous protocol in procedure standards was kept. Both physical therapists were blinded to mode of delivery during the postpartum measurements.

### Statistical analysis

Statistical analysis was performed using SPSS version 26 (SPSS, Chicago, IL, USA). Background and descriptive variables are presented as frequencies with percentages or means with standard deviations (SD). Changes from mid-pregnancy to 12 months postpartum and between examinations postpartum within delivery modes, regarding VRP and PFM strength and endurance, were analyzed using paired-samples *t* test for normally distributed data and Wilcoxon signed rank test for non-normally distributed data. Differences between delivery modes were analyzed by using independent samples *t* test for normally distributed data and Mann–Whitney *U* test for non-normally distributed data. Standard multiple linear regression was used to analyze the influence of demographic and obstetric variables on the recovery of PFM variables at 12 months postpartum to mid-pregnancy. *p* Values < 0.05 were considered statistically significant.

## Results

**Fig. 1:**
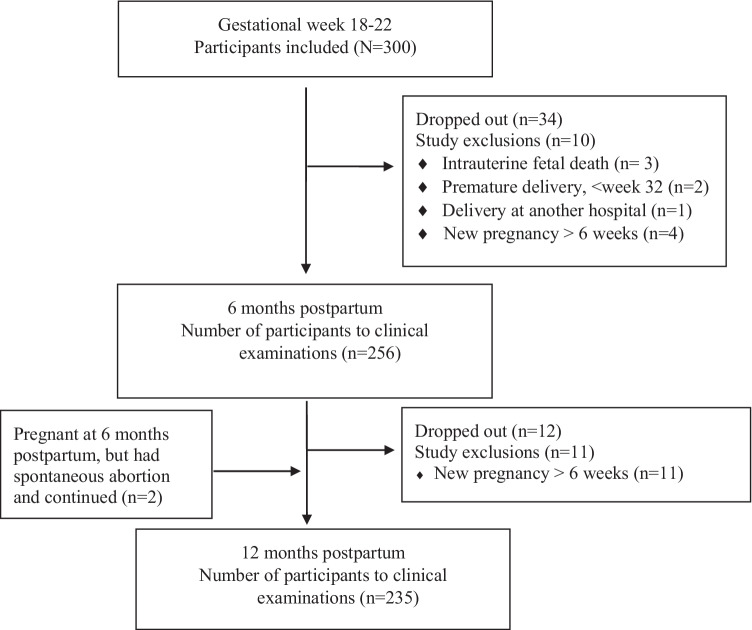
Flowchart illustrating the number of women examined at each clinical examination and the different reasons for non-participation

Two hundred and thirty-five of the 300 nulliparous pregnant women had a complete dataset and provided data for the present study (Fig. 1). Loss to follow-up was 21.7%. Background characteristics of the participants are presented in Table 1.

**Table 1:** Background and obstetric variables of the total study population (*N*=235) and within delivery groups

Variable	Total sample (*n*=235)	NVD (*n*=157)	IVD (*n*=43)	CS (*n*=35)
Background data				
Maternal age (years)	29.6 ± 4.1	29.4 ± 4.1	29.6 ± 3.6	30.4 ± 4.8
Prepregnancy BMI (kg/m^2^)	23.8 ± 3.9	24.0 ± 4.1	23.3 ±3.5	24.0 ± 3.6
BMI 12 months postpartum^a^	24.8 ± 4.4	24.8 ± 4.4	24.3 ± 4.2	25.5 ± 4.9
University or higher education	182 (77.4)	124 (79.0)	35 (81.4)	23 (65.7)
Married or cohabiting	226 (96.2)	149 (94.9)	43 (100.0)	34 (97.1)
Ethnicity white	226 (96.2)	153 (97.5)	41 (95.3)	32 (91.4)
Smoking prepregnancy				
No	175 (74.5)	113 (72.0)	36 (83.7)	26 (74.3)
Sometimes/daily	60 (25.5)	44 (28.0)	7 (16.3)	9 (25.7)
Delivery data				
Total gestational length (days)	280.6 ± 10.7	281.0 ± 10.4	282.5 ±9.1	277.8 ± 14.6
Birth weight (g)	3,496.0 ± 514.1	3,457.0 ± 450.1	3,680.7 ± 507.0	3,443.4 ± 720.5
Head circumference (cm)	35.1 ± 1.6	35.0 ± 1.5	35.7 ± 1.6	35.1 ± 1.9
Vaginal delivery	200	–	–	–
Normal vaginal	157	157	–	–
Instrumental vacuum	39	–	39	–
Instrumental vacuum and forceps	2	–	2	–
Instrumental forceps	2	–	2	–
Cesarean section	35	–	–	35
Acute	27	–	–	27
Elective	8	–	–	8
Length of second stage of labor (min)	71.5 ± 53.0	60.0 ± 41.0	103.0 ± 59.2	162.0 ± 120.8
Epidural (yes)	91 (38.7)	51 (32.5)	26 (60.5)	14 (40.0)
Episiotomy (yes)^b^	64 (31.8)	40 (25.5)	23 (53.5)	1 (2.9)
Perineal tear grade 3 or 4	8 (3.4)	3 (1.9)	5 (11.6)	–
Major levator ani tear 6 weeks postpartum, assessed using transperineal ultrasound	43 (18.3)	24 (15.3)	19 (44.2)	–
Number (*n*) of women unable to perform a correct PFM contraction				
Gestational week 18–22	10 (4.3)	8 (5.1)	–	2 (5.7)
6 months postpartum	7 (2.9)	5 (3.2)	1 (2.3)	1 (2.9)
12 months postpartum	6 (2.6)	4 (2.5)	1 (2.3)	1 (2.9)
Number (*n*) of women who do regular PFMT, >3 times/week				
Gestational week 18–22	35 (14.9)	22 (14.0)	9 (20.9)	4 (11.4)
6 months postpartum	84 (35.7)	57 (36.3)	22 (51.2)	5 (14.7) ^c^
12 months postpartum	21 (8.9)	16 (10.2)	3 (7.0)	2 (5.8) ^d^

Values are given as mean with standard deviation (±SD) and numbers with percentages (%)

*BMI* body mass index, *CS* Cesarean section, *IVD* instrumental vaginal delivery, *NVD* normal vaginal delivery, *PFM* pelvic floor muscle, *PFMT* pelvic floor muscle training

^a^Missing data on 1 participant

^b^Missing data on 34 participants

^c^Missing data on 1 participant

^d^Missing data on 1 participant

### Normal vaginal birth group

At 6 months postpartum, in women who had a normal vaginal birth, the VRP was reduced by 21% (*p*<0.001) and PFM strength by 15% (*p*<0.001) from mid-pregnancy, whereas PFM endurance did not differ. At 12 months postpartum there was a 20% reduction in VRP (*p*<0.001), a 7% reduction in PFM strength (*p*=0.007), and an increase in PFM endurance by 9% (*p*=0.02) compared with mid-pregnancy values (Tables 2, 3). The improvement between 6 and 12 months postpartum in PFM strength and endurance was statistically significant but that in VRP was not (Table 3).

**Table 2:** Vaginal resting pressure (*VRP*), pelvic floor muscle (*PFM*) strength and endurance at mid-pregnancy and postpartum in a sample of nulliparous women with different delivery modes (*N*=235)

Delivery modes	Gestational week 18–22, mean (±SD)	6 months postpartum, mean (±SD)	12 months postpartum, mean (±SD)
NVD (*n*=157)			
VRP (cmH_2_O)	41.9 (±9.6)	32.9 (±7.7)	33.5 (±7.6)
PFM strength (cmH_2_O)	36.2 (±19.6)	30.9 (±19.5)	33.5 (±19.2)
PFM endurance (cmH_2_O/s)	249.3 (±142.6)	244.6 (±163.9)	272.4 (±167.5)
IVD (*n*=43)			
VRP (cmH_2_O)	44.9 (±9.3)	34.7 (±9.0)	35.1 (±8.5)
PFM strength (cmH_2_O)	35.5 (±17.0)	26.8 (±16.0)	30.0 (±18.1)
PFM endurance (cmH_2_O/s)	246.1 (±126.7)	213. 9 (±146.8)	244.1 (±158.4)
CS (*n*=35)			
VRP (cmH_2_O)	44.9 (±12.6)	40.4 (±11.8)	40.7 (±11.7)
PFM strength (cmH_2_O)	37.6 (±17.4)	39.1 (±17.9)	42.2 (±17.7)
PFM endurance (cmH_2_O/s)	263.1 (±131.7)	309.7 (±154.4)	329.8 (±144.6)

Values are given as mean with standard deviation (±SD)

*CS* cesarean section, *IVD* instrumental vaginal delivery, *NVD* normal vaginal delivery, *PFM* pelvic floor muscle, *SD* standard deviation, *VRP* vaginal resting pressure

**Table 3:** Change in vaginal resting pressure (*VRP*), pelvic floor muscle (*PFM*) strength and PFM endurance postpartum and from mid-pregnancy to 12 months postpartum (*N*=235)

Time period variable	Change from mid-pregnancy to 6 and 12 months postpartum				Change between postpartum examinations	
	Gestational weeks 18–22 to 6 months postpartum		Gestational weeks 18–22 to 12 months postpartum		6 months postpartum to 12 months postpartum	
	Mean difference (95% CI)	*p* value	Mean difference (95% CI)	*p* value	Mean difference (95% CI)	*p* value
NVD (*n*=157)						
VRP (cmH_2_O)	−9.0 (−10.2, −7.8)	<0.001	−8.4 (−9.6, −7.2)	<0.001	0.6 (−0.1, 1.3)	0.096
PFM strength (cmH_2_O)	−5.4 (−7.3, −3.5)	<0.001	−2.7 (−4.6, −0.8)	0.007	2.7 (1.6, 3.7)	<0.001
PFM endurance (cmH_2_Osec)	−4.7 (−23.7, 14.4)	0.628	23.1 (3.2, 43.0)	0.023	27.8 (17.7, 37.9)	<0.001
IVD (*n*=43)						
VRP (cmH_2_O)	−10.2 (−12.2, −8.5)	<0.001	−9.6 (−12.0, −7.7)	<0.001	0.3 (−1.0, 1.6)	0.640
PFM strength (cmH_2_O)	−8.7 (−12.5, −4.8)	<0.001	−5.5 (−9.6, −1.3)	0.011	3.2 (1.0, 5.5)	0.006
PFM endurance (cmH_2_Osec)	−32.2 (−64.2, −0.2)	0.049	−2.0 (−34.8, 30.8)	0.904	30.2 (11.9, 48.5)	0.002
CS (*n*=35)						
VRP (cmH_2_O)	−4.4 (−7.0, −1.9)	0.001	−4.1 (−6.7, −1.5)	0.003	0.3 (−1.5, 2.1)	0.723
PFM strength (cmH_2_O)	1.6 (−2.6, 5.8)	0.454	4.6 (0.7, 8.6)	0.023	3.1 (−0.1, 6.2)	0.056
PFM endurance (cmH_2_Osec)	46.7 (10.9, 82.4)	0.012	66.7 (32.7, 100.7)	<0.001	20.0 (−6.6, 46.7)	0.136

The values are presented with mean difference and 95% CI)

Positive values for mean difference, mean increase in measured value for the variable on PFM function

Negative values for mean difference, mean reduced measured value for the variable on PFM function

Paired samples *t* test

*CI* confidence interval, *CS* cesarean section, *IVD* instrumental vaginal delivery, *NVD* normal vaginal delivery, *PFM* pelvic floor muscle, *VRP* vaginal resting pressure

### Instrumental vaginal delivery group

At 6 months postpartum, VRP was reduced by 23% (*p*<0.001) and PFM strength and endurance were reduced by 25% (*p*<0.001) and 13% (*p*=0.049) respectively from mid-pregnancy. At 12 months postpartum VRP was reduced by 21% (*p*<0.001) and PFM strength by 15% (*p*=0.011) when compared with mid-pregnancy values. PFM endurance at 12 months was not different from mid-pregnancy (Tables 2, 3). The improvements between 6 and 12 months postpartum were statistically significant in PFM strength and endurance but not in VRP (Table 3).

### Cesarean section group

Thirty-five women (14.9%) of the present study sample had CS. At 6 months postpartum VRP was reduced by 10% (*p*=0.001) and PFM endurance was increased by 18% (*p*=0.012) compared with mid-pregnancy values. At 12 months postpartum compared with mid-pregnancy measurements, women with CS had a significant reduction in VRP of 9% (*p*=0.003), but a significant increase in PFM strength and endurance of 12% (*p*=0.023) and 25% (*p*<0.001) respectively (Tables 2, 3). There were no statistically significant changes between 6 and 12 months postpartum within the CS group (Table 3).

Table 4 shows the changes in VRP, PFM strength, and endurance between delivery modes from 6 to 12 months postpartum and from mid-pregnancy to 12 months postpartum. There were no statistically significant differences between the normal vaginal delivery group and instrumental vaginal group in any measurements. Statistically significant differences were found between 12 months and mid-pregnancy in VRP, strength, and endurance when comparing CS with normal vaginal delivery and instrumental vaginal delivery respectively.

**Table 4:** Changes in vaginal resting pressure (*VRP*), pelvic floor muscle (*PFM*) strength and PFM endurance between delivery modes from 6 to 12 months postpartum and from mid-pregnancy to 12 months postpartum (*N*=235)

Variable		NVD (*n*=157) vs. CS (*n*= 35)		IVD (*n*=43) vs. CS (*n*= 35)		NVD (*n*=157) vs. IVD (*n*=43)	
		Mean difference (95% CI)	*P* value	Mean difference (95% CI)	*P* value	Mean difference (95% CI)	*P* value
VRP	∆ 6 months pp – 12 months pp (cmH_2_O)	0.3 (−1.4, 2.0)	0.739	−0.01 (−2.1, 2.1)	0.991	−0.3 (−1.8, 1.2)	0.694
	∆ gestational week 18–22–12 months pp (cmH_2_O)	4.3 (1.5, 7.1)	0.003	5.7 (2.5, 9.0)	<0.001	1.5 (−1.1, 4.0)	0.256
PFM strength	∆ 6 months pp–12 mo. pp. (cmH_2_O)	−0.4 (−3.1, 2.3)	0.779	0.2 (−3.5, 3.9)	0.930	0.5 (−1.8, 2.9)	0.647
	∆ gestational week 18–22–12 months pp (cmH_2_O)	7.3 (2.8, 11.8)	<0.001	10.1 (4.4, 15.8)	<0.001	2.8 (−1.5, 7.0)	0.199
PFM endurance	∆ 6 months pp –12 months pp (cmH_2_Osec)	7.8 (−16.9, 32.4)	0.536	10.2 (−20.8, 41.1)	0.515	2.4 (−19.1, 23.9)	0.826
	∆ gestational week 18–22–12 months pp (cmH_2_Osec)	43.6 (−1.4, 88.5)	0.057	68.7 (21.8, 115.5)	0.005	25.1 (−16.5, 66.7)	0.193

Values are presented with mean difference and 95% CIs

PFM strength is calculated as the mean of three maximal voluntary contractions. PFM endurance is reported during a 10-s sustained maximal contraction

Positive values for mean difference indicate greater change in the PFM variable for the former delivery mode in the comparison

Negative values for mean difference indicate minor change in the PFM variable for the former delivery mode in the comparison

*CI* confidence interval, *CS* cesarean section, *IVD* instrumental vaginal delivery, *NVD* normal vaginal delivery, *PFM* pelvic floor muscle *VRP* vaginal resting pressure, ∆ change

Independent-samples *t* test

Table 5 shows results from the multiple linear regression. CS was protective against negative changes in the PFM from mid-pregnancy to 12 months postpartum. There was a statistically significant negative influence of recovery beyond vaginal delivery modes in women with higher BMI at 12 months postpartum, a longer second stage of labor and major tears of the levator ani. Of these factors, the beta coefficient showed that a major tear of the levator ani was the most influential. The degree of negative influence on PFM function set by major levator ani tear (beta = 3.1) was similar to CS having a positive influence (beta = −3.0).

**Table 5:** Variables that impact the recovery of vaginal resting pressure (VRP), PFM strength and PFM endurance at 12 months postpartum (*N*=235)

	Factor					
	∆ VRP GW 18–22–12 months pp, cmH_2_O		∆ PFM strength GW 18–22–12 months pp, cmH_2_O		∆ PFM endurance GW 18–22–12 months pp, cmH_2_O/s	
Variable	B coefficient (95% CI)	*p* value	B coefficient (95% CI)	*p* value	B coefficient (95% CI)	*p* value
CS^a^	−3.0 (−6.0, 0.1)	0.059	−9.7 (−14.7, −4.6)	<0.001	−60.6 (−110.0, −11.2)	0.017
IVD^a^	−0.003 (−2.9, 2.9)	0.998	−2.4 (−7.2, 2.3)	0.314	−18.6 (−65.0, 27.9)	0.431
Age	−0.2 (−0.4, 0.1)	0.101	0.005 (−0.4, 0.4)	0.980	−0.2 (−4.0, 3.6)	0.913
BMI 12 months pp	−0.5 (−0.8, −0.3)	<0.001	−0.4 (−0.8, −0.01)	0.047	−4.5 (−8.1, −0.8)	0.016
Longer second stage of labor	0.003 (−0.02, 0.025)	0.775	0.07 (0.03, 0.1)	<0.001	0.5 (0.2, 0.9)	0.005
Head circumference	−0.2 (−0.9, 0.4)	0.427	0.6 (−0.4, 1.7)	0.235	7.0 (−3.2, 17.2)	0.175
Epidural	0.3 (−1.9, 2.5)	0.783	−3.6 (−7.2, 0.2)	0.051	−32.3 (−67.6, 3.0)	0.073
Major LA tear 6 weeks pp	3.1 (0.4, 5.9)	0.027	8.4 (3.9, 13.0)	<0.001	79.8 (35.3, 124.4)	0.001
Regular PFMT 6 months pp	1.9 (−0.2, 4.0)	0.072	−2.4 (−5.8, 1.1)	0.177	−30.0 (−63.5, 3.6)	0.079

Standard multiple linear regression

*BMI* body mass index, *CI* confidence interval, *CS* cesarean section, *GW* gestational week, *IVD* instrumental vaginal delivery, *LA* levator ani, *pp* postpartum, *PFM* pelvic floor muscle, *PFMT* pelvic floor muscle training, *VRP* vaginal resting pressure, ∆ change

^a^Normal vaginal delivery as reference.

Reported regular PFMT (≥3 times/week) at 6 months postpartum did not influence the results.

## Discussion

The main results of the present study were that there were no negative changes in PFM variables in the CS group, except for VRP, where our results showed a significant reduction of 9% at 12 months compared with mid-pregnancy. For both the normal and the instrumental vaginal groups, VRP and PFM strength had not returned to mid-pregnancy values at 12 months postpartum, with no differences in change between these two groups. BMI at 12 months postpartum, longer second stage of labor and major tears of the levator ani muscle were the only factors that had a negative influence on recovery of the PFM besides delivery mode at 12 months postpartum.

As expected, in the present study there was less negative influence on the PFM in the CS group than in the vaginal delivery groups. Strength and endurance had improved from mid-pregnancy values at 12 months postpartum. However, VRP was still reduced by 9% from mid-pregnancy among women with CS, and this did not improve from 6 to 12 months postpartum. In a study on women aged 49 (SD 12) with POP stage I–III, Brækken et al. [21] found a moderate negative association between LH area and VRP, strength, and endurance. VRP best explained the LH area. VRP may constitute both PFM tension/activation and the area of the LH but the amount of fat, estrogen levels, and vascular factors may also affect this measurement. The results on VRP may therefore be difficult to interpret. Intra- and interrater reliability results are also poorer for VRP than PFM strength and endurance [19], and the results should therefore be interpreted with caution. For future studies we recommend surface EMG as a more exact measure of resting PFM activity [6].

In women with normal and operative vaginal delivery, PFM strength and endurance were reduced both at 6 and 12 months postpartum compared with mid-pregnancy values. However, we found no differences in change of PFM strength and endurance between the normal vaginal group and the instrumental vaginal group. There were few women with forceps in our study, which, together with the sample size for the instrumental delivery group, may explain the lack of statistical significance. It is positive for women’s pelvic health if instrumental vaginal delivery does not negatively affect PFM strength and endurance more than normal vaginal birth. However, in a study of 666 women 6–11 years after birth, PFM strength was reduced by 17 cm H_2_O, *p*<0.001, more after history of forceps delivery than after normal vaginal delivery [22]. In the present study the improvement in PFM strength and endurance continued between 6 and 12 months in both vaginal delivery groups. However, it was still reduced compared with mid-pregnancy variables. Friedman et al. [22] found that women with CS had a PFM strength of 39 cm H_2_O compared with 29 cm H_2_O in women after vaginal delivery (*p*<0.001). Five to 10 years postpartum, Blomquist et al. [5] found that women with at least one vaginal delivery had a lower peak pressure during PFM contraction, defined as <20 cm H_2_O, than women with CS. The reduction in PFM strength and endurance found in the vaginal delivery groups compared with mid-pregnancy values in the present study may be an important finding to guide future clinical practice and follow-up of the PFM in the postpartum period. Blomquist et al. [5] followed 1,143 women, recruited 5–10 years after first delivery, and assessed them annually for PFD up to 9 years with questionnaire, POP-Q, and manometry. They found that, among women who had at least one vaginal delivery, PFM strength of <20 cm H_2_O was associated with a shorter time to event for SUI, overactive bladder, and POP. The associations attenuated when adjusting for genital hiatus and BMI. No association between PFM strength and PFD was found among women who delivered all their children by CS. As the manometers used in their study were different from ours, caution must be used in direct comparisons of results between studies [6, 23].

We have only been able to find one study that has measured PFM variables longitudinally from pregnancy throughout the postpartum period. Elenskaia et al. [9] assessed change in VRP and PFM strength from pregnancy till 12 months postpartum using manometry. They found a significant reduction in VRP and strength 14 weeks postpartum. However, contradictory to the present study, at 12 months, VRP was still reduced, but PFM strength was restored. The reduction in VRP in the Elenskaia et al. [9] study was only 5.1% compared with 20% in our study, and this may also somehow have accounted for the difference in results. Elenskaia et al. [9] did not assess PFM endurance and they did not separate between normal and instrumental vaginal delivery groups. Also, their cohort was different from ours as it also included multiparous women and the loss to follow-up was larger than for our study, with only 39% of the original population attending the last assessment at 12 months. The manometers used were different in size (theirs being much bigger than ours), which has been shown to affect measurements [24]. Furthermore, they had a larger group of women with CS than the present study (23% vs 13%). We do not know the rate of elective CS in their group, but in our study 27 out of 35 (77.1%) had emergency CS, meaning that their PFM may have been stretched and weakened. Elenskaia et al. [9] did not separate women with and without instrumental vaginal delivery and did not assess whether the women had major levator ani tears. The results of the two studies are therefore not directly comparable.

In the present study we found that BMI at 12 months, long stage of labor and major levator ani tears had a negative influence on recovery, with major levator ani tears being the most influential. In the multivariate analysis by Elenskaia et al. [9] two variables remained significant for difference in resting pressure: length of total second stage >60 min and head circumference. For difference in strength, the only significant risk factor was an active second stage greater than 60 min. Head circumference was not a significant factor in our study, but both studies found that second stage of labor had an influence.

Elenskaia et al. [9] found that 37 out of 148 patients (25.0%) who attended both postnatal visits performed PFMT on a regular basis, with no association with PFM variables. Regular training was not further defined in their study but it was defined as ≥3 times/week in the present study. We found that 35.7% and 8.9% reported regular PFMT at 6 and 12 months postpartum respectively. Nevertheless, our results correspond with those of Elenskaia et al. [9], showing no association between reported PFMT and PFM outcome variables. Some of the women in the present study had participated in an RCT with one arm having a 4-month intervention with supervised PFMT group training once a week and everyday PFM exercise at home, but there were no statistically significant differences between exercisers and controls in manometer measurement of PFM function [11]. One explanation of no effect of PFMT in this RCT might be the inclusion of women with diagnosed major levator ani tears. The multivariate analysis of the present study found that major levator ani tears were associated with reduced recovery and we have also reported that women with major levator ani tears have significantly weaker PFM at 6 weeks postpartum [8]. There is a need for further follow-up studies in women with major levator ani tears.

According to a Cochrane review of RCTs of PFMT in the postpartum period, the results vary between studies in effect on both UI and FI and in changes in PFM variables [25]. No effect in some RCTs may be due to natural recovery during the 1st year postpartum, as was shown in many women in the present and in Elenskaia et al.’s study [9]. RCTs of PFMT in women postpartum may therefore need larger sample sizes than in other populations to detect statistically significant and clinically relevant effect sizes. Nevertheless, we found that PFM strength and endurance are not back to mid-pregnancy level at 12 months, and many women may therefore need closer follow-up PFM training than that offered in most health care systems today. Besides delivery mode, degree of major levator ani tears, long second stage, and BMI at 12 months, as was found in our multivariate analysis, may be important factors to consider when planning future health services and RCTs in the postpartum period [26]. Data on breastfeeding or hormonal therapy at 12 months postpartum were not available for this study and may be interesting and important factors to investigate in relation to PFM function in future studies.

Strengths of the present study are the longitudinal prospective design, relatively few losses to follow-up, and the use of reliable and valid measurement methods to assess PFM variables [15, 18] and major levator ani tears [14]. Ability to perform a correct PFM contraction was ascertained by visual observation and vaginal digital palpation, and only measurements with visual inward movement of the catheter/perineum were registered as valid strength assessment [18]. Two trained physical therapists performed the PFM measurements and two trained gynecologists were doing the assessment of major levator ani tears following the protocol of Dietz et al. [12, 13]. Both physical therapists and gynecologists were blinded to birth history during assessments.

Limitations are the use of two assessors for measurement of both PFM variables and major tears. However, both ultrasound and manometer measurements have been tested for inter-tester reliability by our group and have been found to be reliable [14, 19]. Although the loss to follow-up can be considered low (21.7%), this may have influenced the results. However, looking at the reasons for dropping out, the loss to follow-up seems to be unrelated to participation in the study. There was no power calculation for change in PFM variables, and reduction in sample size when grouping the participants according to mode of birth may have reduced the ability to detect statistically significant results. On the other hand, differences in PFM variables have been found between groups of this size, and statistically significant differences were found between many variables. The results of the present study might be used for power calculation in the planning of future studies. We did not have any data on PFM variables before mid-pregnancy. This would be ideal but is difficult to achieve. Most women in the cohort were white and 76.4% had university or college education. The generalizability of the results can therefore only be done to this group of women. There is a need for larger studies including multiethnic cohorts in the peripartum period.
